# Identification and Function of Exchange Proteins Activated Directly by Cyclic AMP (Epac) in Mammalian Spermatozoa

**DOI:** 10.1371/journal.pone.0037713

**Published:** 2012-05-25

**Authors:** Alvaro Miro-Moran, Isaac Jardin, Cristina Ortega-Ferrusola, Gines M. Salido, Fernando J. Peña, Jose A. Tapia, Ines M. Aparicio

**Affiliations:** 1 Cell Physiology Research Group, University of Extremadura, Caceres, Spain; 2 Laboratory of Spermatology, Veterinary Teaching Hospital, University of Extremadura, Caceres, Spain; Universidade Federal do Rio de Janeiro, Brazil

## Abstract

The role of cAMP in spermatic functions was classically thought to be mediated exclusively through the activation of Protein Kinase A (PKA). However, it has recently been shown that cAMP also exerts its effects through a PKA-independent pathway activating a family of proteins known as Epac proteins. Therefore, many of the spermatic functions thought to be regulated by cAMP through the activation of PKA are again under study. We aimed to identify and to investigate the role of Epac proteins in spermatozoa using a specific permeable analog (8-Br-2′-O-Me-cAMP). Also, we aimed to study its relationship with E-cadherin, an adhesion protein involved in fertility. Our results demonstrate the presence and sub-cellular distribution of Epac 1 and Epac 2 in mammalian spermatozoa. Capacitation and the acrosome reaction induced a change in the localization of Epac proteins in sperm. Moreover, incubation with 8-Br-2′-O-Me-cAMP prompted an increase in Rap1 activation, in the scrambling of plasma membrane phospholipids (necessary for the capacitation process), the acrosome reaction, motility, and calcium mobilization, when spermatozoa were incubated in acrosome reaction conditions. Finally, the activation of Epac proteins induced a change in the distribution of E-cadherin. Therefore, the increase in the acrosome reaction, together with the increase in calcium (which is known to be essential for fertilization) and the Epac nteraction with E-cadherin, might indicate that Epac proteins have an important role in gamete recognition and fertilization.

## Introduction

Freshly ejaculated spermatozoa, when deposited in the female tract, undergo numerous changes and modifications, which are prompted by the hormonal and chemical composition of the fluid female tract, that confer the ability to fertilize the oocyte. The first event observed in the spermatozoa is a disruption of the asymmetrical distribution of membrane phospholipids at the anterior sperm head and a cholesterol efflux leading to the elevation of intracellular calcium (Ca^2+^) and bicarbonate (HCO_3_^−^) [1], [2], [3]. Both ions stimulate an atypical adenylyl cyclase present in sperm, which is called soluble adenylyl cyclase (sAC) [4], . The activation of AC leads finally to an increase in the intracellular concentration of cAMP, which in turn activates protein kinase A (PKA), a serine/threonine kinase. The activation of cAMP/PKA leads to downstream events associated with capacitation, motility hyperactivation, and the acrosome reaction (AR) [6], [7], [8].

Several years ago, the role of cAMP in spermatic functions was thought to be mediated exclusively through the activation of protein kinase A (PKA). However, it has been shown that cAMP also exert its effects through a PKA-independent pathway activating a family of proteins known as Epac proteins [9], [10]. Epac is an acronym for the exchange proteins activated directly by cyclic AMP, a family of cAMP-regulated guanine nucleotide exchange factors (cAMPGEFs). Two isoforms of Epac, namely Epac 1 (RAPGEF3) and Epac 2 (RAPGEF4), have been identified so far, both of which couple cAMP production to the activation of Rap, a small molecular weight GTPase of the Ras family [11]. Epac 1 and Epac 2 are products of independent genes: Epac 1 a protein comprising 881 amino acids (molecular mass 100 kDa), whereas Epac 2 is a protein comprising 1011 amino acids (molecular mass 110 kDa). Epac1 and Epac2 are multi-domain proteins consisting of an N-terminal regulatory region and a C-terminal catalytic region. The N-terminal regulatory domain bears a disheveled, Egl-10, pleckstrin (DEP) domain implicated in membrane association and a high-affinity cAMP-binding domain (cAMP-B). Epac2 additionally contains a second low-affinity cAMP-A domain of uncertain biological function. A Ras exchange motif (REM) domain acts as an intramolecular bridge between the regulatory and the catalytic regions to stabilize the guanine nucleotide exchange (GEF) domain. Epac proteins also bear a Ras-associating (RA) domain, which is present in several Ras-interacting proteins. A CDC25 homology domain (CDC25HD) in the C-terminal catalytic domain exhibits GEF activity for Ras-like GTPases [9]. X-ray crystallography studies of full-length Epac2, solved in the absence of cAMP, indicate the presence of autoinhibitory properties in the C-terminal region, which cease upon binding of cAMP [12]

In somatic cells, the role of Epac has been extensively documented, as it is involved in a wide range of functions. While Epac 1, through the activation of Rap1, has been reported to participate in cell adhesion, cell-cell junction, cell differentiation, and inflammatory processes, among others (reviewed in [13]), Epac 2, in pancreatic beta cells, mediates cAMP-induced insulin secretion [14] and calcium mobilization [15]. However, scarce literature about Epac can be found in germinal cells. Epac 1 has recently been identified in ejaculated human and stallion sperm [16], [17] and in epididymal mouse sperm, [18], while Epac 2 has been detected in mouse spermatogenic cells [19] and ejaculated stallion sperm [17]. Using AMPc analogs that are cell permeant and selective activators of Epac, it has been observed that these drugs are able to induce an increase in the AR [16], [17], [20], regulate the conversion of microtubule sliding into flagellar bending [21], or modulate the membrane potential in mammalian spermatozoa [17]. Furthermore, the cAMP-Epac-Rap1 pathway was also proven to have a role in spermiogenesis by Aivatiadou et al. [22]. They showed that the generation of transgenic mice expressing an inactive Rap1 mutant in differentiating spermatids resulted in the anomalous release of immature spermatids within the lumen of seminiferous tubuli. The underlying mechanism was probably related to the impairment of germ-Sertoli cell contacts, which was correlated with the expression of VE-cadherin, an adhesion molecule the function of which in cell-cell contacts in somatic cells is known to be regulated by cAMP/Epac/Rap1 [22].

Therefore, with the importance of cAMP in the regulation of capacitation, motility, and the AR in mind, it is important to reexamine the regulation of these processes that were previously thought to occur exclusively through the activation of PKA. Therefore, we aimed to identify and localize Epac proteins and Rap1, which is the direct downstream effector of Epacs, on mammalian spermatozoa. Using a membrane-permeant, metabolically activatable stimulator of Epac (8-Br-2′-O-Me-cAMP), we studied the scrambling of plasma membrane phospholipids, the AR, motility, and intracellular Ca^2+^. Finally, we tested the possible relation of Epacs with the epithelial cadherin (E-cadherin), an adhesion molecule that has been shown to be involved with human fertility [23].

## Results

### Identification and subcellular distribution of Epac 1 and Epac 2 in unstimulated mammalian spermatozoa

**Figure 1:**
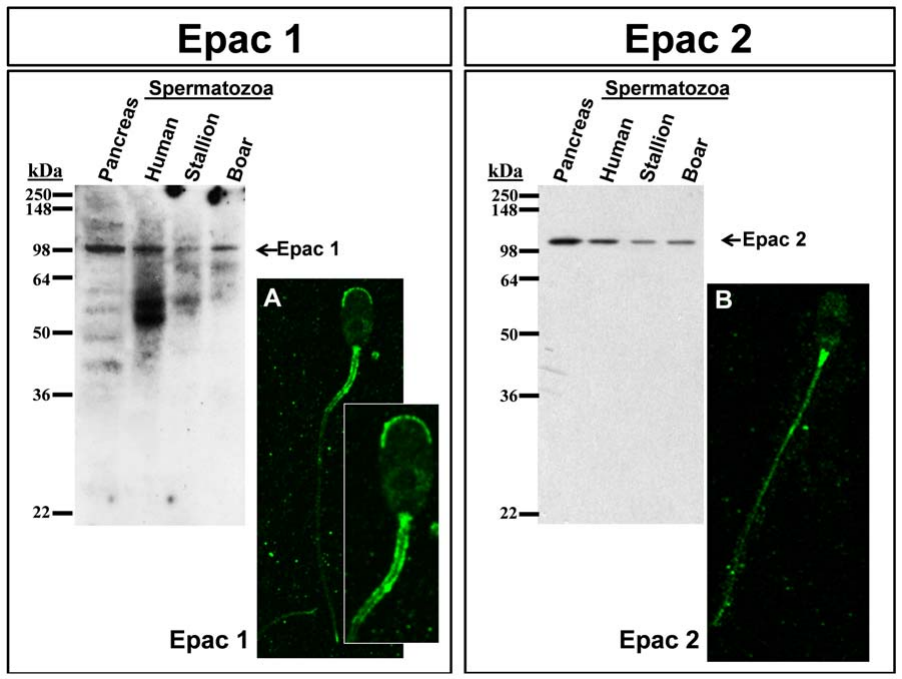
Identification and subcellular distribution of Epac 1 and Epac 2 proteins in mammalian spermatozoa. Twenty µg of proteins from rat pancreas lysates and from human, stallion and boar spermatozoa lysates were resolved by SDS-PAGE, followed by Western blotting with anti-Epac 1 or anti-Epac 2 antibodies, as described in Materials and Methods. Immunolocalization was performed as described in Materials and Methods using specific antibodies against Epac 1 and Epac 2 proteins. A: Epac 1 immunolocalization with representative areas digitally augmented; B: Epac 2 immunolocalization.

We investigated the presence of Epac 1 and Epac 2 in spermatozoa with specific commercially available antibodies (Abs) by Western blotting and indirect immunofluorescence (Fig. 1). Sperm cell lysates from human, stallion and boar spermatozoa were used in the immunoblotting study. To avoid possible individual differences, these lysates were obtained from pooled samples from at least three different individuals Moreover, to accurately evaluate the immunoblotting results, sperm samples were used in parallel with a lysate from rat pancreas, in which the presence of Epac 1 and 2 have previously been shown [24], [25]. Results showed that the anti-Epac 1 polyclonal Ab (Ref: SAB1100714) recognized a single band of approximately 100 kDa in all samples, indicating the presence of this protein in mammalian spermatozoa (Fig. 1, left panel). These results were corroborated by an additional, unrelated antibody (Epac 1 monoclonal Ab from Cell Signaling, Ref: 4155), which gave virtually identical results. The Epac 1 Abs that have shown immunoreactivity in the immunoblotting were subsequently used to examine, by indirect immunofluorescence, the subcellular localization of the target protein in permeabilized boar spermatozoa. Epac 1 was localized in the apical region of the acrosome and in the membrane of the midpiece in the tail on boar spermatozoa, being the immunofluorescence much more diffuse and less intense in the rest of the tail (Fig. 1A).

Identical approaches to those above were followed to investigate the presence of Epac 2 in sperm cells. Anti-Epac 2 monoclonal Ab (Ref: WH0011069M1) recognized a single band of approximately 110 kDa in all samples, also indicating the presence of this protein in mammalian spermatozoa (Fig. 1, right panel). The results were also assessed with an additional antibody (Epac 2 monoclonal Ab from Cell Signaling, Ref: 4156), which also rendered similar results. Subsequently, we investigated the subcellular location of Epac 2 in boar spermatozoa with the Abs used in the immunoblotting study. Results showed that the immunofluorescence was localized mainly in the connecting piece in boar spermatozoa; it was weakly distributed along the mid-, principal, and end pieces of the tail and apparently absent in the head (Fig. 1B).

### Immunolocalization of Epac 1/2 in boar spermatozoa under non-capacitating and capacitating conditions

**Figure 2:**
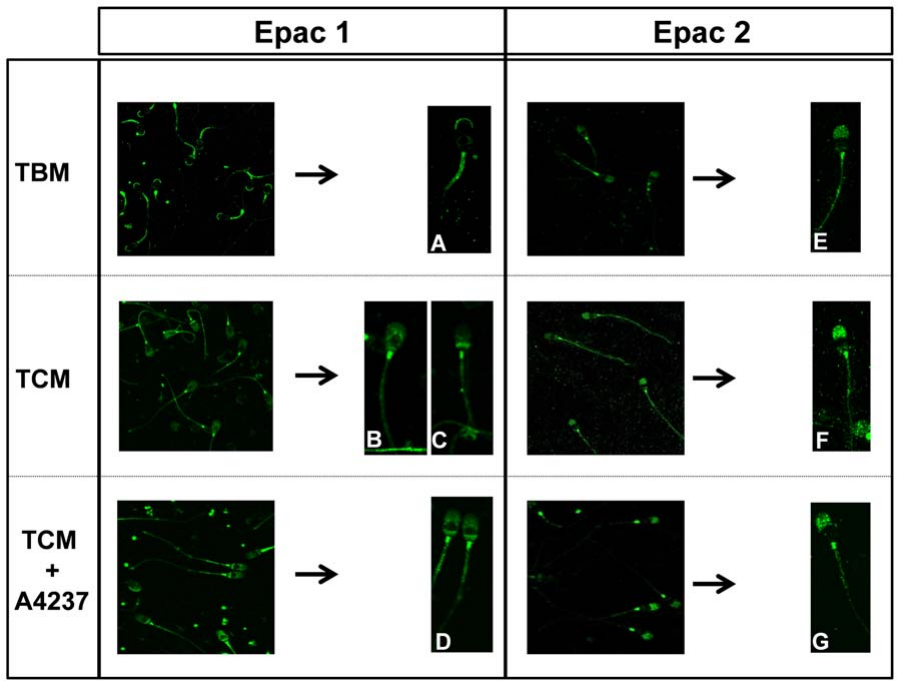
Immunolocalization of Epac 1 and Epac 2 proteins in boar spermatozoa under non-capacitating and capacitating conditions. Immunolocalization was performed as described in Materials and Methods using specific antibodies against Epac 1 and Epac 2 proteins in spermatozoa previously incubated in TBM, TCM and TCM+A23187.. Representative images of spermatozoa showing Epac 1 localization in TBM (A), TCM (B, C) and TCM+A23187 (D) and Epac 2 localization in TBM (E), TCM (F) and TCM+A23187 (G). N = 3 replicates.

As indicated earlier, in non-capacitating conditions (TBM), Epac 1 was localized in the apical region of the acrosome and in the middle piece of the spermatozoa (Fig. 1A; Fig. 2A). However, the incubation of spermatozoa in capacitating conditions (TCM) produced a clear change in the localization of Epac 1 showing two different patterns of distribution (Fig. 2B; Fig. 2C). The first pattern observed which was detected in a higher percentage of spermatozoa, consisted of the accumulation of fluorescence in the connection piece between the tail and head (Fig. 2B). The signal from the second pattern was localized to the post-acrosome region and also to the connecting piece (Fig. 2C). Finally, a signal corresponding to Epac 1 in acrosome-reacted spermatozoa (TCM+A23187) was extended all over the head (except in the equatorial segment) and again in the connecting piece (Fig. 2D).

In non-capacitating conditions, Epac 2 was mainly located in the connecting piece of the spermatozoa and weakly to the tail (Fig. 1B; Fig. 2E). However, when spermatozoa were incubated in capacitating conditions (TCM), a signal was also detected in the acrosome region (Fig. 2F), where the labeling was even stronger when the AR was induced with A23187 (Fig. 2G).

### Effect of the Epac activation on the scrambling of plasma membrane phospholipids of boar spermatozoa

**Figure 3:**
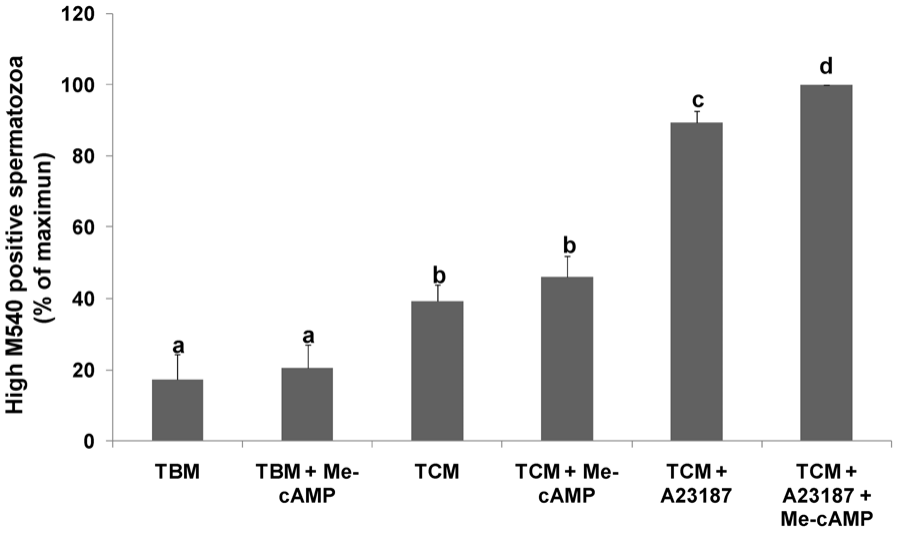
Effect of the Epac activation on the scrambling of plasma membrane phospholipids of boar spermatozoa. The scrambling of plasma membrane phospholipids was monitored by flow cytometry by using M540 (1.3 µM). Spermatozoa were also stained with YO-PRO-1 (25 nM) to distinguish viable and non-viable cells. Results are represented as the percentage of maximum of viable cells recorded with high M540 ± SEM. Columns with different letters indicate significant differences (P<0.05). N = 5 replicates.

One of the phenomena that occurs during capacitation is the change observed in the lipid membrane order [1]. The scrambling of plasma membrane phospholipids can be monitored by merocyanine-540 (M540), which has been used previously as a probe to monitor bicarbonate activation in individual cells [26], [27]. Therefore, scrambling of plasma membrane phospholipids was assessed by flow cytometry using double staining with M540 and YO-PRO-1; the latter was used to distinguish between viable and non-viable cells. The incubation of spermatozoa in TBM for 2 hours in the presence of Me-cAMP (50 µM) did not induce any detectable change in the membrane architecture of the cells. However, when cells were incubated in TCM, a significant increase was observed in the percentage of viable spermatozoa stained with high M540 compared to TBM. This increase observed was not modified in the presence of Me-cAMP (Fig. 3). Finally, induction of AR by incubation of spermatozoa in TCM in the presence of A23187 (10 µM) produced a significant increase in viable cells stained with high M540, and this increase was significantly higher in the presence of Me-cAMP (50 µM) (Fig. 3).

### Effect of Epac activation on the acrosomal status of boar spermatozoa

Acrosomal status was evaluated by flow cytometry using FITC-PNA, as it has been demonstrated to be an accurate probe for studying boar sperm AR [28]. To distinguish between viable a non-viable cells, we used propidium iodide simultaneously with PNA.

**Figure 4:**
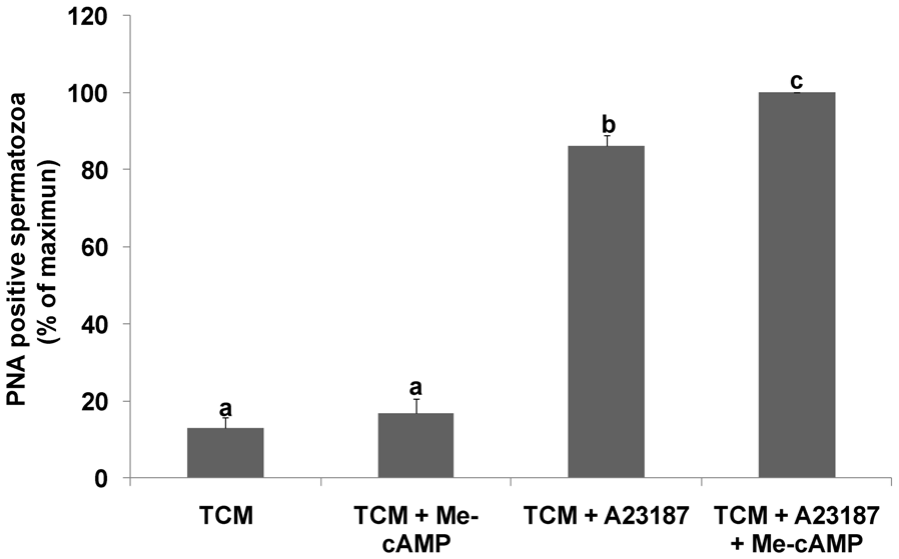
Effect of Epac activation on the acrosomal status of boar spermatozoa. Acrosomal status was evaluated by flow cytometry using FITC-PNA (0.5 mg/ml) and IP (1.2 mM), which stains non-viable cells. Results are represented as percentages of maximum of viable spermatozoa recorded as PNA positive cells ± SEM. Columns with different letters indicate significant differences (P<0.05). N = 5 replicates.

The incubation of spermatozoa in TCM in the presence of Me-cAMP has no effect on the percentage of viable cells positive for PNA compared to TCM alone. The induction of AR by addition of A23187 (10 µM) significantly increased the percentage of viable cells labeled with PNA compared to TCM alone, and this increase was significantly higher in the presence of Me-cAMP (Fig. 4), indicating that Epac proteins likely play a positive role in the induction of the AR.

### Role of Epac activation on sperm motility

**Figure 5:**
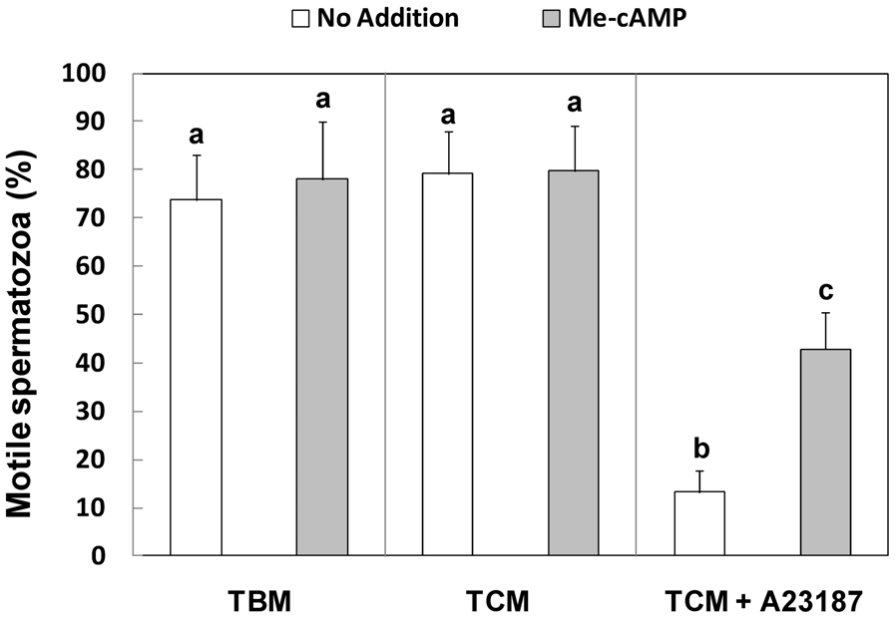
Effect of Epac activation on boar spermatozoa motility. The motility of spermatozoa, incubated in TBM, TCM and TCM+A23187 and in the presence or absence Me-cAMP was assessed by ISAS. Graphics show the percentage of motile spermatozoa. Columns with different superscripts are statistically different from each other, so that for (a–b–c) (P<0.05). N = 5 replicates.

The incubation of boar spermatozoa in TBM or TCM in the presence or absence of Me-cAMP showed no significant differences in the percentage of motile spermatozoa. As previously described [29], the induction of the AR caused a significant reduction in the percentage of motile spermatozoa. However, when the AR was induced in presence of Me-cAMP (50 µM), the percentage of motile cells was significantly higher than the percentage of motile cells incubated in TCM plus A23187 alone (Fig. 5), indicating that the activation of Epac proteins plays a role in the regulation of sperm motility during the AR.

### Role of Epac activation on the intracellular calcium concentration

Taking into account the increase in the AR, a Ca^2+^-dependent exocytotic process induced in presence of the Epac activator (Fig. 4), we postulated that this could indicate the influence of Epac proteins in Ca^2+^ mobilization in sperm. This hypothesis was supported by previous reports in somatic cells, indicating that Epac proteins play a role in the regulation of activity of Ca^2+^ channels [30]. Therefore, we aimed to investigate the possible role of Epac activation in the intracellular calcium mobilization.

**Figure 6:**
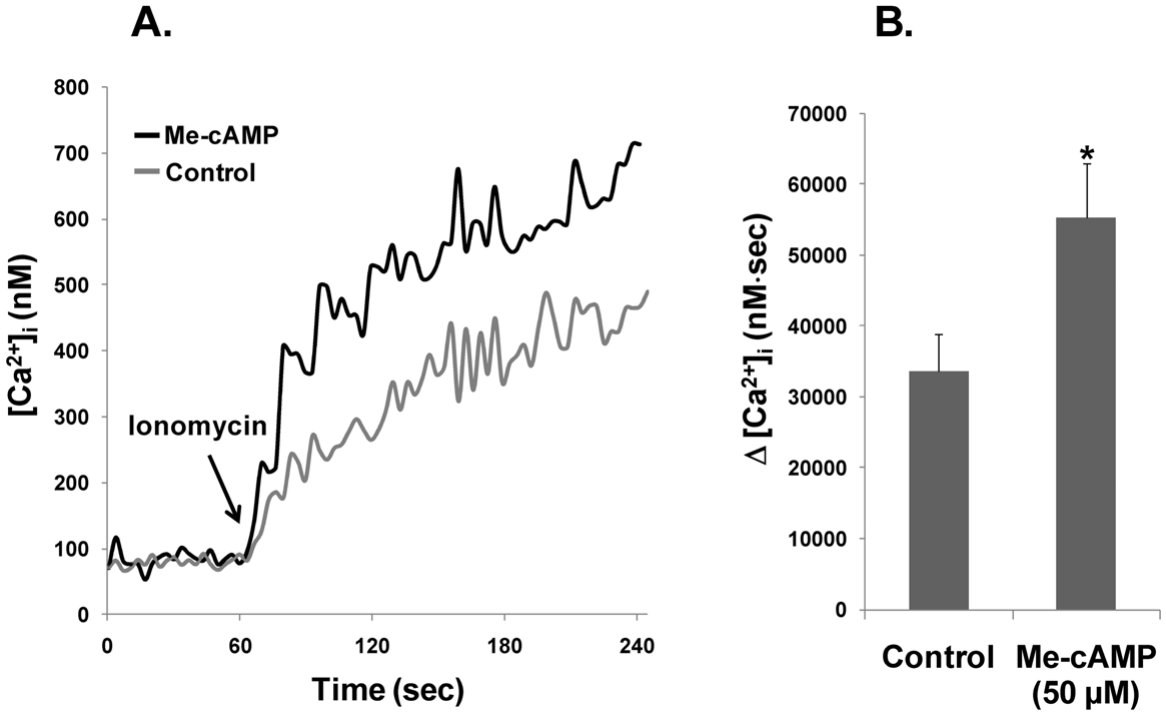
Measurement of cytosolic free calcium concentration ([Ca^2+^]_i_). Samples were incubated in TCM for 90 min in the presence or absence of Me-cAMP (50 µM). Then, cells were incubated in TCM with 2 µM for 30 min, and washed in Na-HEPES. Fluorescence of FURA-2-AM was recorded using a fluorescence spectrophotometer (Varian Ltd., Madrid, Spain), and changes in [Ca2+]i were monitored every second. A) [Ca2+]i values were calculated and expressed as nM. Traces are representative of 5 independent experiments. B) Results of the integral of the rise in [Ca2+]i of 5 independent experiments ± SEM. Column marked with * indicate significant differences compared to control (P<0.05). N = 7 replicates.

In TCM, media that contains Ca^2+^, no differences in the [Ca^2+^]_i_ were seen between the control and spermatozoa treated with Me-cAMP (Fig. 6). The addition of ionomycin after 60 sec resulted in a transient increase in [Ca^2+^]_i_ in both the control and Me-cAMP-treated cells, being the rise of [Ca^2+^]_i_ significantly higher in the cells treated with Me-cAMP (Fig. 6A). This effect was also clearly seen when considering the integral of the rise in [Ca^2+^]_i_ for 180 sec after the addition of ionomycin, which was significantly higher in Me-cAMP-treated cells compared to control cells (33654.35±5125.22 versus 55269.02±7741.97 nM⋅sec, respectively) (Fig. 6B).

### Rap1 identification and activation

**Figure 7:**
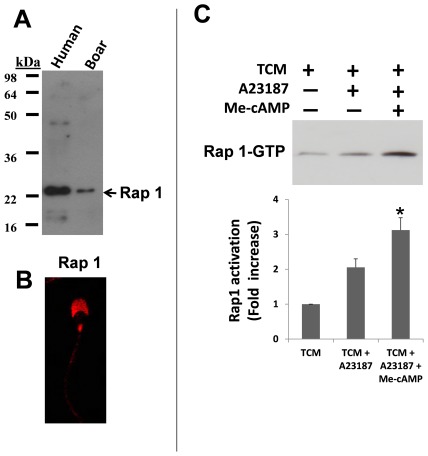
Rap1 identification and activation. Twenty µg of proteins from human and boar spermatozoa lysates were resolved by SDS-PAGE, followed by Western blotting with anti-Rap1 antibody (7A), as described in Materials and Methods. To study the immunolocalization of Rap1, boar spermatozoa were incubated overnight with specific anti-Rap1 antibody (7B). Rap1 activation was studied using a commercial kit according to the manufacturer's protocol. Western blotting was performed using an anti-Rap1 antibody, as described in Materials and Methods (7C). Graphic shows the fold-increase of Rap-GTP with respect to the control (TCM alone) ± SEM. N = 4 replicates.

Next, we determined whether Rap1, a direct downstream effector of Epacs, is expressed in boar spermatozoa using human sperm as a positive control [20]. Anti-Rap1 antibody detected a single band corresponding to the molecular weight expected for this protein in boar spermatozoa lysates (∼22 kDa) (Fig. 7A). The same antibody was used to assess the localization of Rap1, showing that this protein was mainly expressed in the acrosome region and in the connecting piece of boar sperm (Fig. 7B)

Once we observed that Rap1 was present in spermatozoa, our next aim was to study Rap1 activation in samples incubated in TCM and TCM plus A23187 with or without Me-cAMP. The induction of the AR (TCM plus A23187) produced an small increase in Rap1 activation compared to capacitating conditions (TCM), although it was not statistically significant. However, the induction of the AR in samples containing Me-cAMP resulted in a statistically significant increase in Rap1 activation (Fig. 7C)

### Effect of Epac activation in the distribution of E-cadherin

With the previously shown results in mind, in which the [Ca^2+^]_i_ was increased in the presence of Me-cAMP, we next investigated the distribution of E-cadherin, a Ca^2+^-dependent cell adhesion molecule regulated by Rap1 [31] and involved in the processes of gamete recognition and fertilization [23]

**Figure 8:**
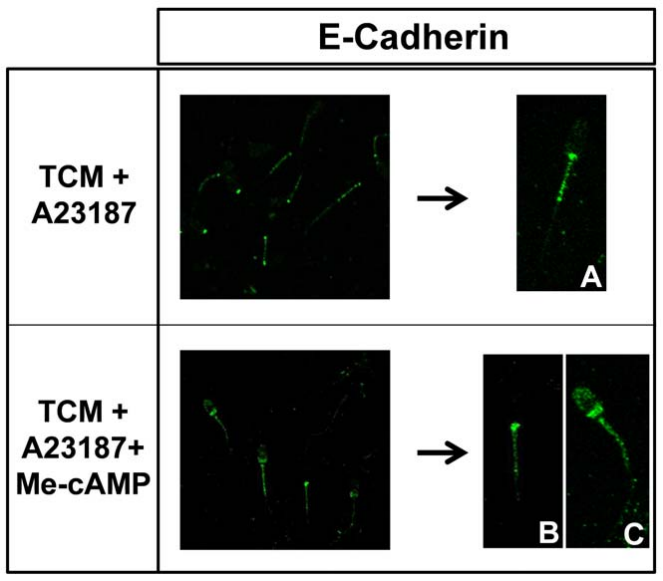
Effect of Epac activation on the distribution of E-cadherin. To study the immunolocalization of E-cadherin, boar spermatozoa were fixed and incubated overnight with a specific antibody and then were washed and incubated with the appropriated secondary antibody, which was labeled with Alexa 488. Representative images of spermatozoa showing E-cadherin localization in: TCM+A23187 (A) and TCM+A23187+Me-cAMP (B).

In TCM plus A23187 (10 µM) -incubated cells, E-cadherin was detected mainly in the connecting and middle pieces of the tail (Fig. 8A). The addition of Me-cAMP (50 µM) produced a change in the distribution of E-cadherin in acrosome-reacted cells (TCM+A23187), in which the immunofluorescence was detected in the middle pieces of the tail and the head, which was a stronger signal in the post-acrosomic region of the spermatozoa (Fig. 8C). This pattern of distribution was observed in 80% of spermatozoa, while 20% of cells presented the pattern described previously for those spermatozoa incubated in TCM plus A23187 without the cAMP analog (Fig. 8B).

### Colocalization of Epac 1 and E-cadherin

As the localization of E-cadherin changed after the activation of Epac proteins with Me-cAMP (Fig. 8C) and the pattern observed was similar to the Epac 1 distribution in acrosome-reacted spermatozoa (Fig. 1D), we aimed to study the colocalization of both proteins when the AR is induced in either the presence or absence of Me-cAMP.

**Figure 9:**
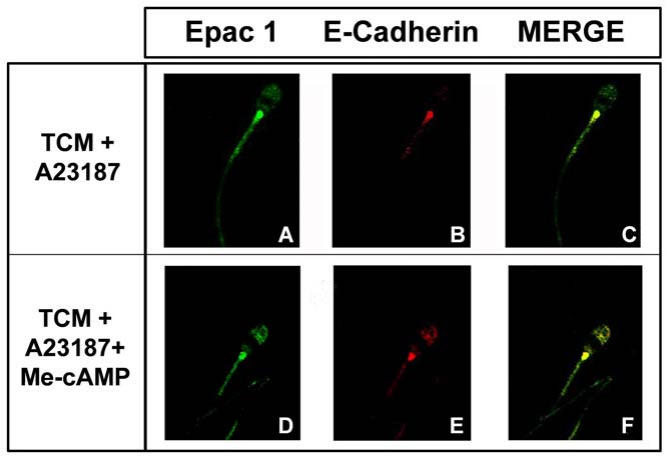
Colocalization of Epac 1 and E-cadherin. To study the co-localization of Epac 1 and E-cadherin, boar spermatozoa were incubated overnight with specific antibodies (anti Epac1 and E-cadherin) and, then, were washed and incubated with the appropriated secondary antibodies, which were labeled with Alexa 488 and Alexa 568. Representative images of spermatozoa showing Epac 1 (A), E-cadherin (B), and colocalization of both proteins (C) in TCM+A23187. Representative images of spermatozoa showing Epac 1 (D), E-cadherin (E), and colocalization both proteins (F) in TCM+A23187+Me-cAMP (50 µM). N = 3 replicates.

In a medium inducer of the AR without Me-cAMP, the colocalization of Epac1 (Fig. 9A) and E-cadherin (Fig. 9B) was observed in the connecting and midpiece of the tail (Fig. 9C).

When the AR was induced in the presence of the Me-cAMP (50 µM), Epac 1 localization was unchanged (Fig. 9D), while E-cadherin (Fig. 9E) distribution changed completely after Epac activation, as shown previously in Figure 8C. In the merged slide (Fig. 9F), Epac1 and E-cadherin colocalized in the acrosome and post-acrosome regions in the head and in the connecting and midpiece in the tail of the sperm (Fig. 9F).

## Discussion

cAMP is a second messenger that is essential for many biological processes. Since the discovery of the cAMP target Epac, many of the spermatic functions known to be regulated by cAMP through the activation of PKA should be redefined.

In somatic cells, cAMP analogs that selectively activate Epac have helped to reveal a role for Epac in processes ranging from insulin secretion to cardiac contraction and vascular permeability [10]. The role of cAMP through PKA in boar spermatozoa has been extensively documented [6], [7], [8]. Here, we show for the first time the identification and the function of cAMP through Epac/Rap1 in the scrambling of plasma membrane phospholipids, the AR, motility, and [Ca^2+^]_i_ mobilization. Moreover, we demonstrate the possible interaction of Epacs with E-cadherin signaling, an important membrane-bound protein involved in fertilization [23].

Immunolocalization of Epac 1 in boar spermatozoa has been shown to be localized in the membrane overlying the acrosome, in agreement with previous reports [17], and in the membrane of the middle piece of the tail. Epac 2 has recently been demonstrated to be localized to the membrane overlying the midpiece of both stallion and mouse sperm [17], whereas in boar spermatozoa this protein was mainly localized to the connecting piece. The differences found in the localization of Epac proteins, although with big similarities, might be due to the species studied.

Similar to previous studies in mouse spermatogenic cells, in which stimulated cells revealed the translocation of endogenous Epac 2 [19], the incubation of mature ejaculated spermatozoa in stimulating conditions produced a change in the localization of Epac 1, masking the signal observed overlying the acrosome in basal conditions and detected in the postacrosomic and acrosomic regions in capacitated and acrosome-reacted spermatozoa. These are known to be fusogenic areas in gamete fusion, [32] indicating the possible role of Epac 1 in the AR. However, although a higher intensity of the signal was detected in the acrosome region in stimulating conditions, Epac 2 localization pattern remained mainly unchanged compared to unstimulated cells. Therefore, the different pattern found in basal conditions together with the change observed in the distribution under stimulating conditions might be pointing to a different function for each isoform. Nevertheless, further studies are clearly required to clarify this hypothesis.

Once we identified Epac isoforms, we studied the possible role of Epacs in spermatic functions, including the scrambling of plasma membrane phospholipids, phenomena associated with capacitation, the AR, and motility. During capacitation, phospholipid scrambling induces the exposure of endogenous aminophospholipids at the surface of anterior acrosomal region of the head of the plasma membrane [33]. This induces a concomitant increase in membrane fluidity resulting in protein and lipid redistribution and the efflux of cholesterol [27]. These lipid rearrangements have been shown to coincide with enhanced sperm–zona-binding capacity [34] and the induction of the AR [35]. In our study, the specific analog 8-Br-2′-O-Me-cAMP induced an increase in scrambling of plasma membrane phospholipids, the AR, and [Ca^2+^]_i_ mobilization. Therefore, taking all these results together, the increase in the scrambling of plasma membrane phospholipids observed might be involved in the increase in [Ca^2+^]_i_ and the subsequent increase in the AR [35], which is known to be a Ca^2+^-dependent process. However, our results could be also explained through the model suggested by Braham et al. in human sperm (2006), as they also observed an increase in the AR and Ca^2+^ mobilization using an analog that specifically activates Epacs. In this model, AR is organized as a bifurcated pathway, with two separate limbs that diverge downstream of cAMP/Epac. The end point of one limb (Rap-PLC) is the mobilization of intracellular Ca^2+^, whereas the other (Rab3A-α-SNAP/NSF-SNAREs) assembles the fusion protein machinery so that the outer acrosomal and plasma membranes become physically connected. Both pathways operate in a concerted fashion and converge at or downstream of intracellular Ca^2+^ mobilization to accomplish exocytosis; once Ca^2+^ is released into the cytosol, the protein machinery senses the rise in concentration, and the whole system evolves to open the fusion pores [16]. However, although this model is in harmony with our results, it cannot explain why the effect of Epac/Rap1 activation in boar spermatozoa was observed only in capacitating media and in the presence of the A23187 ionophore. The same results were observed in stallion sperm, in which the addition of 8pCPT (a specific analog of cAMP that activates Epacs) induced acrosomal exocytosis only in capacitating conditions. Therefore, these results could indicate the possible intervention of some other event that occurs during capacitation or the AR that is necessary for the participation of Epacs through Rap1 activation. One plausible explanation is the change in intracellular distribution observed in Epacs under stimulating conditions. The translocation of Epacs might be completely necessary for its interaction with other proteins and, therefore, for triggering a proper signaling cascade, as occurs with PKA and its interaction with AKAP proteins in the control of spermatic functions [36], [37]. Moreover, this hypothesis is supported by previous findings in mouse spermatogenic cells, in which activated Epac 2 translocates from the cytosol where it resides when not stimulated to the Golgi region where Rap1 is located, demonstrating the effective Rap1-Epac 2 protein interaction and therefore exerting its action, among others, through Rap-activated Rho GTPase-activating Protein (RA-RhoGAP) to promote the progression of spermatogenesis [19].

It is well known the importance of cAMP/PKA in the regulation of mammalian spermatozoa functions [6], [7], [8]. However, to the best of our knowledge, no studies have addressed the role of the cAMP/Epac in the regulation of motility parameters in sperm. According to previous studies in human and boar sperm [29], [38], A23187 induced a significant decrease in sperm motility, indicating a negative effect of Ca^2+^ in this function. The drop in motility after treatment with A23187 has been associated with calcium's effect on cellular ATPases [39]. Ca-ATPases are located in the plasma membrane and are important in maintaining low intracellular levels of calcium by pumping calcium out of the cell. When cells are treated with A23187, there is a massive influx of calcium into the sperm, which may be too great for the calcium-ATPase to equilibrate to a normal [Ca^2+^]_i_. However, although we showed that the selective analog Me-cAMP mobilized [Ca^2+^]_i_, it mitigated the effects of A23187 on sperm motility. In somatic cells, Epac proteins regulate Rap-1 sensitive sarco-endoplasmic reticulum Ca^2+^-ATPases (SERCAs). Although, there is no endoplasmic reticulum (ER) or Golgi apparatus in mature sperm cells, which are usually known to act as internal Ca^2+^ stores in somatic cells, the presence of SERCA2 has been demonstrated in human, bovine, and murine spermatozoa. SERCA2 was detected in human as well as in murine and bovine sperm in the acrosome and midpiece areas. SERCA2 was also observed in the midpiece in human sperm [40]. Hence, taking into account those comments above, it is possible that the increase in motility observed after activation of Epac in acrosome reacting conditions might be due to the higher activation of Ca^2+^-ATPases, which would prompt a decrease in [Ca^2+^]_i_ by pumping Ca^2+^ out of the cell. The increase in motility observed after Epac activation could be explained with those shown previously in hamster spermatozoa, in which the Epac/Rap pathway regulates the conversion of microtubule sliding into flagellar binding [21].

Since the discovery of cAMP/Epac intracellular signaling in mammalian spermatozoa and its possible involvement in the regulation of spermatic functions, no studies have addressed the role of this signaling machinery in fertilization or the proteins involved in gamete recognition. Multiple studies have been performed to identify the molecules responsible for the binding and fusion of gamete membranes in the fertilization process. Recently, accumulating data points to the possible involvement of cell adhesion molecules, such as cadherins, in gamete recognition and fertilization [23], [41]. Cadherins are cell surface glycoproteins involved in Ca^2+^-dependent cell-cell adhesion [42], which is regulated by the Epac/Rap1 intracellular pathway [31]. Therefore, taking into account the results in the present work, in which Epac is involved in the AR and [Ca^2+^]_i_, we aimed to study the possible interaction between Epac and E-cadherin proteins. When cells were incubated in presence of Me-cAMP, there was a change in the localization of E-cadherin, which resulted in a colocalization with Epac 1 in acrosome-reacted spermatozoa treated with Me-cAMP. Therefore, as a relocalization of E-cadherin during capacitation seems to be necessary for its subsequent participation in adhesion events during human fertilization [23], our results indicate the possible involvement of Epac1/Rap1 activation in these events through the relocalization of E-cadherin, as the localization of Epac1 did not changed after its activation with the specific analog, Me-cAMP.

To summarize, the current work shows for the first time the presence and localization of Epac 1, Epac 2, and Rap1 in boar spermatozoa and its participation in the scrambling of plasma membrane phospholipids, which are necessary for capacitation process, and in the AR. Moreover, Epac is involved in the mobilization of intracellular Ca^2+^ and in the relocalization of E-cadherin. As the effects of Epac were observed during the AR, it seems to indicate that this protein participates in this process, causing a redistribution of Epacs having as a consequence the Epac interaction with other proteins such us Ca^2+^ channels. Moreover, the fact that the Epac/Rap1 effect is observed at late stages during the AR together with the increase in Ca^2+^ [30] (which is known to be essential for fertilization) and with its interaction with E-cadherin, might indicate that Epac has an important role in gamete recognition and fertilization.

## Materials and Methods

### Materials

8-Bromo-2′-O-methyladenosine-3′,5′-cyclic monophosphate sodium salt (8-Br-2′-O-Me-cAMP or Me-cAMP) was purchased from Biolog (Bremen, Germany). A23187 was purchased from Calbiochem (La Jolla, CA). Fura-2-AM, YO-PRO-1, *Arachis hypogaea* (peanut) agglutinin FITC-conjugated (FITC-PNA), propidium iodide (PI), anti-mouse and anti-rabbit IgG secondary antibodies (Abs) labeled with the fluorescent probe Alexa 488, and anti-rabbit IgG secondary Ab labeled with Alexa 568 were from Invitrogen (Eugene, OR). Protease inhibitor cocktail tablets (Complete mini, EDTA-free) were from Roche (Basel, Switzerland). Anti-Epac 1 polyclonal Ab (Ref: SAB1100714) and anti-Epac 2 monoclonal Ab (Ref: WH0011069M1) were from Sigma-Aldrich (St. Louis, MO). A different set of Abs against Epac 1 (monoclonal Ab, Ref: 4155) or Epac 2 (monoclonal Ab, Ref: 4156) were from Cell Signaling (Beverly, CA). Anti E-cadherin monoclonal Ab was from BD Biosciences-Pharmingen (Franklin Lakes, NJ). Anti-Rap1 polyclonal Ab (sc-65) was from Santa Cruz Biotechnology (Santa Cruz, CA). Tris/glycine/SDS buffer and Tris/glycine buffer were from Bio-Rad (Hercules, CA). The active Rap1 pull-down and detection kit, anti-mouse and anti-rabbit IgG (HRP)-conjugated secondary Abs and enhanced chemiluminescence detection reagents were from Pierce (Rockford, IL). Nitrocellulose membranes were from Schleicher & Schuell (Keene, NH).

### Media

For porcine spermatozoa, a Tyrode complete medium (TCM) was used as sperm-capacitating media, consisting of 96 mM NaCl, 4.7 mM KCl, 0.4 mM MgSO_4_, 0.3 mM NaH_2_PO_4_, 5.5 mM glucose, 1 mM sodium pyruvate, 21.6 mM sodium lactate, 0.5 mM CaCl_2_, 10 mM NaHCO_3_, 20 mM HEPES (pH 7.45), and 3 mg/ml of BSA. The TCM was equilibrated with 5% CO_2_. A non-capacitating variant of TCM was made by omitting BSA, CaCl_2_, and NaHCO_3_ and was noted as Tyrode basal media (TBM). Both media were prepared on the day of use and were maintained at an osmolarity of 290–310 mOsm/kg at pH 7.45 and 38°C. Human semen was washed in RPMI-1640 media (Sigma-Aldrich, St. Louis, MO) followed by dilution in Na-HEPES solution containing 140 mM NaCl, 4.7 mM KCl, 1.1 mM CaCl_2_, 10 mM glucose and 10 mM HEPES (pH adjusted to 7.4 with NaOH). Stallion semen was diluted and washed with a commercially available extender (INRA 96; IMV Technologies, L'Aigle, France). These procedures were reviewed and approved by the Animal Ethics Committee of the University of Extremadura and were performed in accord with Spanish and European guidelines for research on animals.

### Collection and washing of semen

Boar semen samples from Duroc boars of proven fertility were purchased (Semen Porcino Andalucia SL, Sevilla, Spain) as commercial artificial insemination (AI) doses diluted to 30×10^6^ sperm cells per ml in 80 ml of a commercial extender (MR-A, Kubus, Madrid, Spain). In all experiments, seminal doses from up to four animals were pooled to minimize individual boar variation, using semen from at least eight boars. Doses were stored at 17°C before use. Seminal doses were then mixed and centrifuged for 3 min at 2300 g and washed with TBM. Samples of 1.5 ml containing 100×10^6^ spermatozoa/ml were then incubated at 38°C in TCM or TBM for 2 h. Induction or the AR was carried out by using 10 µM of the ionophore A23187 for 10 min in spermatozoa already incubated for 110 min in TCM at 38°C. Incubations were performed for 2 h in the presence or absence of 8-Br-2′-O-Me-cAMP (50 µM). 8-Br-2′-O-Me-cAMP (Me-cAMP) is an analog of the natural signal molecule cyclic AMP in which the hydrogen in position 8 of the heterocyclic nucleobase is replaced by bromine and the 2′-hydroxy group has been methylated. The free 2′-ribose hydroxyl group in cyclic AMP is essential for stimulation of cAMP-dependent protein kinase (PKA); the methylated structure of 8-Br-2′-O-Me-cAMP-AM is an extremely poor PKA activator and allows for specific discrimination between both signaling pathways. In addition, the polar phosphate is masked by an acetoxymethyl group.

Human semen was obtained from four men (20–40 years old), as approved by the institutional review board and the ethics committee of the University of Extremadura, as well as in accordance with the Declaration of Helsinki. Each subject was confirmed to be in good health by means of their medical histories and a clinical examination including routine laboratory test and screening. The subjects were all non-smokers, were not using any medications, and abstained from alcohol. Informed consent was obtained from all the participants. Samples were collected early in the morning by masturbation after 4–5 days of sexual abstinence and allowed to liquefy for 30 minutes (37°C, 5% CO_2_) before processing. Semen was washed twice in RPMI-1640 media (250 g; 10 min). The supernatant was then discarded, and the pellet was resuspended in Na-HEPES as previously described [43].

Stallion semen was obtained from four Andalusian horses individually housed at the Veterinary Teaching Hospital of the University of Extremadura, Spain. The stallions were maintained according to the institutional and European regulations. Semen was collected with the use of a Missouri model artificial vagina with an in-line filter lubricated and warmed from 45 to 50°C. The semen was carried immediately to the laboratory where was washed and diluted 1∶1 in INRA 96 extender as previously described [44]. To avoid interferences of external proteins with the identification of intrinsic proteins, samples were finally washed twice in Na-HEPES.

### Western blotting

To separate the proteins according to their apparent molecular masses, SDS-PAGE was performed according to Laemmli [45]. Samples (1 ml) were washed with Phosphate-uffered saline (PBS), resuspended in lysis buffer (50 mM Trizma base, 150 mM NaCl, 1% Triton X-100, 1% deoxycholate, 1 mM EGTA, 0.4 mM EDTA) supplemented with a protease inhibitor cocktail and phosphatase inhibitor (0.2 mM Na_3_VO_4_), and sonicated for 5 s at 4°C. The homogenate was centrifuged for 15 min at 10,000 g at 4°C, and the supernatant containing soluble proteins in non-ionic and ionic detergents was used for further protein analysis. Proteins were denatured by boiling for 5 min at 95°C in a loading buffer supplemented with 5% mercaptoethanol. The protein content was calculated using the Bradford assay [46]. Twenty micrograms of protein extract of spermatozoa was loaded and resolved by SDS-PAGE on a 10% polyacrylamide gel. The proteins were then transferred to a nitrocellulose membrane, which was subsequently blocked with blocking buffer [5% nonfat dry milk, in a Tris-buffered saline Tween-20 (TBST) containing 10 mM Trizma base, 100 mM NaCl and 0.05% Tween 20] for 1 h at room temperature. Immunoblotting was performed by incubating the membranes in blocking buffer overnight at 4°C with Epac 1 or Epac 2 antibodies diluted at 1/1,000. Membranes were then washed in TBST and incubated with the appropriate species-specific horseradish peroxidase-conjugated secondary antibodies for 45 min at room temperature (RT). Following 3 washes for 10 min each with TBST, the signal was visualized using a SuperSignal West Pico Chemiluminescent Substrate Kit according to the manufacturer's instructions. Band intensity was quantified using the software Scion Image for Windows, version 4.02 (Scion Corp., Frederick, MD).

### Indirect Immunofluorescence and Confocal Microscopy

To study the subcellular localization of proteins, boar spermatozoa were washed and resuspended in PBS, adjusting the cell concentration to 10×10^6^ cells/ml. Fifteen microliters of sperm suspension was spread on poly-L-lysine-coated slides and allowed to attach for 10 min. Spermatozoa were then fixed with 4% formaldehyde in PBS for 15 min at room temperature and permeabilized with 0.2% Triton X-100 (v/v) in PBS for 5 min. Slides were washed three times for 10 min with PBS and incubated in PBS supplemented with 5% BSA (w/v) for 90 min to block nonspecific sites. After blocking, slides were incubated with anti-Epac 1, anti-Epac 2, and anti-E-cadherin antibodies overnight at 4°C diluted 1/100 in PBS containing 5% BSA (w/v). The following day, samples were extensively washed with PBS and further incubated for 45 min at room temperature with the appropriate secondary antibody diluted to 1/500 in PBS containing 5% BSA (w/v), consisting of an anti-mouse IgG antibody conjugated with the Alexa 488 fluorescent dye (Molecular Probes). Finally, the slides were thoroughly washed with PBS and examined with a Bio-Rad MRC1024 confocal microscope. The samples were excited at 488 nm with an argon laser, and their emissions were recorded using a 515-nm long-pass filter set. The absence of nonspecific staining was assessed by processing the samples without primary antibody. Transmission of light was also recorded using a different photomultiplier tube.

### Motility analysis

Spermatozoa were incubated in TBM or TCM in the presence or the absence of Me-cAMP (50 µM) for 2 h at 38°C. The AR was further induced by addition of A23187 (10 µM) for 10 min. After incubation, 2 µl of the samples diluted at 50×10^6^ spermatozoa/ml were loaded in 20-µm-deep Leja chambers (Leja, Nieuw-Vennep, The Netherlands), placed in a thermostatized microscope stage (37°C). Analysis was based on the examination of 25 consecutive digitized images obtained from a single field using a 20× negative-phase contrast objective. Images were taken with a time lapse of 1 s; the image capture speed was therefore once every 40 ms. The number of objects incorrectly identified as spermatozoa was minimized on the monitor by using the playback function. With respect to setting parameters for the ISAS program (Projectes i Serveis R+D, SL; Buñol, Spain), an object with an average path velocity (VAP) <10 mm/s was considered immobile, while objects with a velocity ≥10 mm/s were considered motile.

### Flow cytometry

For flow cytometry analysis, spermatozoa were incubated in TBM or TCM in the presence or absence of Me-cAMP (50 µM) for 2 h at 38°C. The AR was further induced by the addition of A23187 (10 µM) for 10 min. To analyze the scrambling of membrane phospholipids, the samples, each containing 50×10^6^ spermatozoa/ml, were labeled with merocyanine-540 (M540) (1.3 µM) and YO-PRO-1 (25 nM) for 10 min at room temperature. For the determination of acrosomal integrity, the samples were labeled with peanut agglutinin FITC-conjugated (FITC-PNA) (0.5 mg/ml) and propidium iodide (PI) (1.2 mM) for 5 min at room temperature. The samples were analyzed on a FACScan flow cytometer (Becton Dickinson, San Jose, CA). The system collects fluorescence data in the logarithmic mode and light scatter data in the linear mode. Only sperm-specific events, which appeared in a typically L-shaped scatter profile, were positively gated. For each file, 10,000 events were stored in the computer and only live spermatozoa (propidium iodide/YO-PRO-1-negative) were further analyzed with Cell Quest software (Becton Dickinson, San Jose, CA).

### Measurement of intracellular free calcium concentration ([Ca^2+^]_i_)

Samples were incubated in TCM for 90 min in the presence or absence of Me-cAMP (50 µM). Then, cells were incubated in TCM with Fura-2-AM (2 µM) for 30 min. Finally, the spermatozoa were washed in TCM, and 1 ml of sample was loaded into a quartz cuvette. Fluorescence was recorded using a fluorescence spectrophotometer (Varian Ltd., Madrid, Spain) with excitation wavelengths of 340 and 380 nm and emission at 505 nm, and changes in [Ca^2+^]_i_ were monitored every second. After 1 min, ionomycin (10 µM) was added to the cellular suspension. [Ca^2+^]_i_ values were calculated according to the Grynkiewicz method [47] and expressed as nM⋅sec.

### Rap1 activation assay

Boar spermatozoa were washed and incubated in TCM in the presence or absence of Me-cAMP (50 µM) for 110 min. The AR was further induced by the addition of A23187 (10 µM) for 10 min. Samples were lysated, and the protein concentration was measured with a Bradford assay. Six hundred micrograms of total protein was loaded per sample. Rap1 activation was detected using a commercial kit (Active Rap1 Pull-Down and detection kit, Pierce) according to the manufacturer's protocol.

### Statistical analysis

Data were analyzed using ANOVA followed by Scheffe's t-test. All statistical analyses were carried out using the SPSS version 15.0 software package for Windows (SPSS Inc., Chicago, IL, USA). Differences between groups were considered significant when P-values were less than 0.05.
